# Successful retransplantation in a patient with acute primary graft failure

**DOI:** 10.1186/1749-8090-8-S1-O301

**Published:** 2013-09-11

**Authors:** M Urban, J Pirk, O Szarszoi, I Netuka

**Affiliations:** 1 Department of Cardiac Surgery, Institute for Clinical and Experimental Medicine, Prague, Czech Republic

## Background

Cardiac retransplantation in the setting of primary graft failure has been traditionally associated with dismal results. We report a successful outcome of a patient who suffered acute graft failure and was initially rescued with short term mechanical assist support and subsequently retransplanted.

## Methods and results

Case report of 56 year old male with end stage heart disease on the background of ischemic cardiomyopathy bridged to transplantation witha continuous-flow mechanical assist device. After 125 days of support with HeartMate II he received a cardiac allograft from 53 year old male who died from intracranial hemorrhage. After unremarkable procedure with bicaval technique with a total 83 minutes of cold ischemia we observed poor contractility during initial attempt at weaning from CPB. Severe acute predominantly left ventricular failure was confirmed on transesophageal echocardiography. After sixty minutes of reperfusion together with inotropic, vasodilator and inhaled nitric oxide we made the second unsuccessful attempt at weaning and finally resolved to rescue the patient with short-term LVAD Levitronix CentriMag. After stabilization another donor heart became available 16 days after the first transplantation. The second graft came from a 35 year old female who succumbed to embolic stroke. The retransplantation (ischemic time 82 minutes) went uneventfully and the patient was eventually discharged. He is now four years out of surgery in clinically good condition with good graft function.

## Conclusions

Primary graft failure after the heart transplantation is a catastrophic complication with gloomy prognosis. Growing experience with mechanical circulatory support devices and improvements in peritransplant care has made the salvage of even these critically ill patients possible. Given the huge financial and human cost involved as well as scarcity of donor organs the decision to proceed with retransplantation has to be based on careful patient selection and subject to vigorous multidisciplinary validation.

